# Investigation of the effect of carnitine on cerebral vasospasm in experimental subarachnoid hemorrhage model

**DOI:** 10.1038/s41598-023-50025-3

**Published:** 2023-12-27

**Authors:** Gokhan Resitoglu, Mehmet Yigit Akgun, Ozkan Ates, Mustafa Namik Oztanir

**Affiliations:** 1 Department of Neurosurgery, Karaman Research and Education Hospital, Karaman, Turkey; 2 Department of Neurosurgery, Koc University Hospital, Istanbul, Turkey; 3 Department of Neurosurgery, Bezm-i Alem University, Istanbul, Turkey

**Keywords:** Neuroscience, Neurology

## Abstract

The vasospasm, which develops after subarachnoid hemorrhage (SAH), is an unenlightened table in terms of etiology and results. It is usually associated with decreased perfusion, which is associated with decreased blood flow distal to the affected artery and can be demonstrated radiologically. Acetyl-L-carnitine (ALCAR) can be found in brain tissue and easily crosses the blood–brain barrier. Therefore, in this study, we aimed to investigate the therapeutic efficacy of ALCAR, which is an effective antioxidant amine, on vasospasm development after experimental SAH. In our study, 35 adults male Wistar RATs weighing between 235–250 g were used. These RATs were divided into five groups with n = 7. Group 1 Control group, Group 2 SAH + SF (carrier solution), Group 3 SAH + ALCAR 50 mg\kg intraperitoneally, Group 4 SAH + ALCAR 100 mg\kg intraperitoneally and Group 5 SAH. Subarachnoid hemorrhage was induced by giving autologous arterial blood to the cisterna magna of the animals in groups 2, 3, 4, and 5. At 0.-12.- 24.- 36.- 48.- 60. and 72. h, Group 2 was injected with SF, Group 3 with intraperitoneally ALCAR 50 mg\kg, and Group 4 with intraperitoneally ALCAR 100 mg\kg, respectively. Following perfusion and fixation, the animals were subjected to a wide craniectomy, and the brain, cerebellum, and brain stems were removed globally. Then, sections were taken from the basilar arteries of all animals and photographed at 40X magnification. Basilar artery lumen cross-sectional areas, basilar artery areas, and wall thicknesses were measured from these sections. The basilar artery lumen cross-sectional area was found to be significantly larger in the groups in which SAH was formed and ALCAR 50 mg\kg and ALCAR 100 mg\kg were given compared to the group with only SAH and SAH + SF (p = 0.0408). Basilar artery wall thickness increased in all groups except the control group (p < 0.05). In light of all these findings, it was concluded in our study that Carnitine was effective in the resolution of vasospasm in the experimental SAH model.

## Introduction

Spontaneous subarachnoid hemorrhage (SAH); is generally defined as bleeding that occurs due to an aneurysm rupture and develops in the subarachnoid space filled by the cerebrospinal fluid (CSF)^1,2^. In general, there is no consensus on the incidence due to the presence of various risk factors such as different lifestyles, genetic makeup, smoking, alcohol, hypertension, substance use, and oral contraceptive use. It has been reported that the annual incidence of spontaneous SAH varies between 10–16/100,000^3^. SAH is the cause of 5–10% of all bleeding strokes^4^. The overall mortality rate of this condition is 30–70%, and severe neurological disorders are seen in 10–20% of survivors^5^. Rebleeding and vasospasm are mainly responsible for the high mortality and morbidity rates.

Cerebral vasospasm is defined as a delayed and irreversible ischemic neurological deficit that occurs after subarachnoid hemorrhage and progresses with the narrowing of the cerebral arterial caliber^6,7^. It is usually associated with decreased perfusion distal to the affected artery and can be demonstrated radiologically and remained the most important complication affecting mortality and morbidity in patients with SAH^8^. The severity of cerebral vasospasm is related to the amount of blood in the subarachnoid cavity^9^. Cerebral vasospasm can be examined under two main categories as angiographic and symptomatic vasospasm. Angiographic vasospasm is usually present in the 3-5th day of SAH. The time when arterial lumen contraction is determined to be maximum is between 5 and 14 days of bleeding. And between 2 and 4 weeks, vasospasm is expected to resolve gradually^10^. Symptomatic vasospasm is seen only in 20–30% of cases and its timing is parallel to angiographic vasospasm^11^. In the presence of symptoms such as neurological deficit, confusion or aphasia within 3–4 days following the bleeding, vasospasm should be suspected. Subsequently, cortical ischemia and epileptic seizures may be seen. Although very different findings may occur depending on the severity of the spasm, the first neurological finding is usually diplopia^12^.

There is no adequate drug in every aspect that can solve the vasospasm and protect the brain from ischemia and infarcts. However, generally accepted and partially effective treatment options such as hypervolemia, hypertension, and hemodilution therapy can be used.^13^.

Carnitine (3-hydroxy-4-N-trimethylaminobutyrate); is a vitamin-like quaternary amine that is similar in structure to amino acids and it is usually found in our body in the form of L-isoform. While 75% is taken in the diet, 25% is synthesized from the essential amino acids like lysine and methionine in many organs, especially in the liver, kidney, and brain^14^. It has three derivatives: L-Carnitine, Acetyl-L-carnitine (ALCAR), and Propionyl-L-Carnitine. ALCAR is the acetyl ester of carnitine. It can be found in brain tissue and easily crosses the blood–brain barrier. It provides Acyl- CoA to the tricarboxylic acid cycle (TCA) in mitochondria. Here, along with oxidative phosphorylation and energy transport chain, it provides the consumption of excess oxygen. Thus, the O2 concentration and the production of reactive oxygen derivatives (ROS) are reduced, thereby fulfilling its antioxidant role^15^. In our study, we aimed to investigate the therapeutic efficacy of ALCAR, which we know has antioxidant activity, in the vasospasm period developed after SAH created in the experimental model.

## Materials and methods

This study was approved by Inonu University Experimental Animals Ethics Committee with decision number 2012/A-54. Rats were obtained from Inonu University Experimental Animal Production and Research Center and the experimental part of the study was carried out at Inonu University Medical Faculty Experimental Animal Production and Research Laboratory. All animals received humane care in compliance with the principles of laboratory animal care developed by the National Academy of Sciences. All methods are reported in accordance with ARRIVE guidelines. Also, animal procedures were performed according to the “Guide for the Care and Use of Laboratory Animals” principles.

Thirty-five adult Wistar male rats weighing 235–250 g, healthy and not used in any previous study, were used. The animals were housed in a standard laboratory environment with constant temperature, a 12-h light/dark cycle, and 55–60% humidity. During the experiments, the animals were subjected to standard feeding, housing, and care conditions. Subjects were randomized into five groups of seven subjects each. In this study, control group (no SAH) (n = 7), SAH performed (n = 7, n = 6 at the end of the study), ALCAR (50 mg/kg/12 h) with SAH (n = 7, n = 6 at the end of the study)), SAH performed with ALCAR (100 mg/kg/12 h) (n = 7 at the end of the study, n = 6) and SAH performed with placebo [(carrier molecule, 0.9% saline SF), (n = 7, n = 6 at the end of the study)]. The study started on 35 Wistar Albino adult male rats in 5 main groups, and the study was completed with 31 rats.

O-Acetyl-L-carnitine HCl (Sigma-Aldrich, Product No: A6706) was used in our study. Drug doses were prepared by calculating ALCAR amounts and dissolving them in physiological saline as 5 mg in 0.1 ml. Anesthesia of the experimental animals was achieved by intraperitoneal injection of a mixture of Ketamine Hydrochloride (60 mg/kg) (Ketalar, Parke Davis) and Xylazine Hydrochloride (10 mg/kg) (Rompun-2% Bayer) in spontaneous respiration 12 h before fasting. Additional dose anesthesia was performed with Xylazine when necessary. During the experiment, the body temperature of the rats was controlled with a rectal temperature probe and kept constant at 37 °C. After the study, the rats were left at normal room temperature (20–22 °C). After providing anesthesia, all rats were shaved between the inion and the atlas, and approximately 2 cm skin area ​​ was prepared. Then, the femoral artery was catheterized and 0.3 ml of nonheparinized arterial blood was collected. The head was hyperflexed and the cisterna magna was entered with a PPD injector from the atlanto-occipital distance. An equal amount (0.2 ml) of CSF was drained from the subjects in the experimental group. Again, to the same subjects, an equal amount (0.2 ml) of nonheparinized blood taken from the femoral artery was injected slowly^16,17^. After the rats were fully awakened, they were taken into their cages. A second cisterna magna puncture was performed at the 48th hour in the experimental group, and nonheparinized blood was injected^16^.

**Figure 1:**
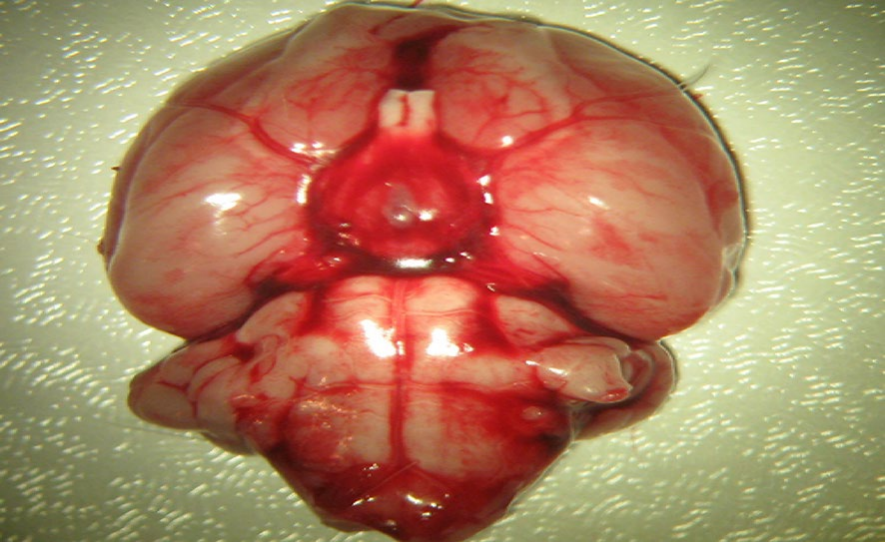
Subarachnoid hemorrhage the subject's brain, cerebral and brain stem from the bottom, removed in one piece.

**Figure 2:**
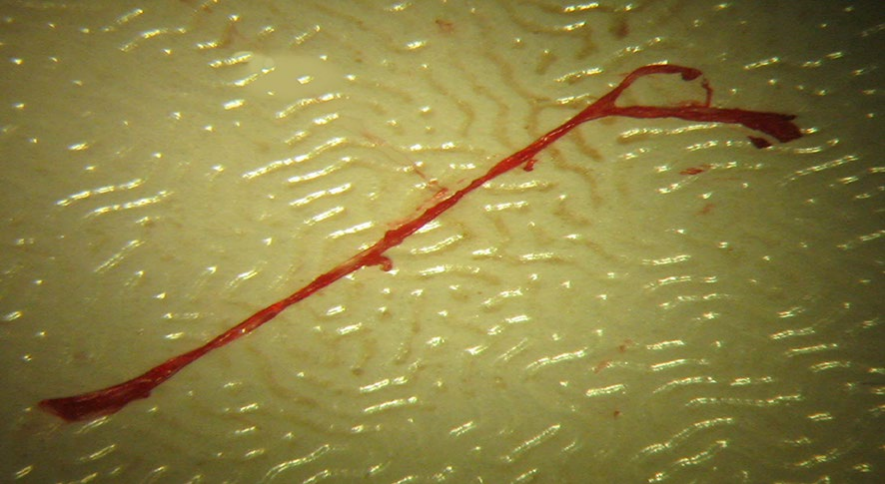
The basilar artery dissected from the basilar sulcus under the microscope.

At the end of 72 h, all subjects were anesthetized by IP injection of a mixture Ketamine Hydrochloride (60 mg/kg) (Ketalar, Parke Davis) and Xylazine Hydrochloride (10 mg/kg) (Rompun-2% Bayer) in spontaneous respiration, and then all subjects underwent thoracotomy^18^. An average of 6–7 cc of blood was taken from all subjects by entering the left ventricle. Following the blood collection procedure, intracardiac saline was given for 5 min, and the brain tissue was purified from blood elements. Macroscopically, diffuse subarachnoid hemorrhage was observed around the vertebral arteries and basilar artery on the basal surface of the brainstem in all subjects who underwent SAH (Fig. 1). The basilar artery was dissected and removed from the sections passing through the brain stem under the microscope (Fig. 2). The basilar artery sections were fixed in glutaraldehyde, and the sections taken from the brain stem and brain tissue were fixed in 10% formaldehyde. Samples taken for the preparation of tissue homogenates were stored in aluminum foil at -30 ^0^C. The stored preparations were treated with 1/10 sodium phosphate buffer (Na2HPO4/NaH2PO4) of 0.9 ml pH:7 of 0.1 g brain tissue to obtain homogenate. Then, it was homogenized at 12.000 rpm for 2 min using a Teflon-coated homogenizer in a glass tube placed in an ice container. This homogenate was then centrifuged at 3500 *rpm* at + 4 °C for 15 min. Obtained supernatants were used for ADMA (Asymmetric Dimethyl Arginine), SDMA (Symmetrical Dimethyl Arginine) and Nitric Oxide (NO) measurements. Histological sections stained with hematoxylin–eosin, transverse sections of basilar arteries and surrounding nerve tissue were evaluated histopathologically by using light microscope. Rats were sacrificed with an overdose of intraperitoneal sodium thiopental (100 mg/kg).

Ensuring animal welfare in experimental research is essential both ethically and scientifically. We give them the ability to have a suitable shelter that protects against both physical and emotional discomfort, the ability to enjoy and engage in behaviors and activities that are considered natural for the species, and the ability to experience an emotionally healthy environment.

### Statistical analyses

All statistical analyses were performed using IBM SPSS 20.0 software (IBM Corp., Armonk, NY, USA). Data are reported as the mean ± SD for normally distributed continuous variables and as number and percentage for dichotomous variables. Since the assumption of normal distribution was not provided, the Kruskal–Wallis test was used to compare the median of the groups. Multiple comparisons were made with Connover's analysis of variance. A two-tailed p < 0.05 was considered to indicate significant differences.

### Ethical approval

This study was approved by Inonu University Experimental Animals Ethics Committee with decision number 2012/A-54. Rats were obtained from Inonu University Experimental Animal Production and Research Center and the experimental part of the study was carried out at Inonu University Medical Faculty Experimental Animal Production and Research Laboratory.

## Results

### Macroscopic findings

After bilateral total craniectomy, in Group 2, Group 3, Group 4 and Group 5 patients in whom subarachnoid hemorrhage was created by autologous blood injection into the cisterna magna, a clot was detected along with diffuse subarachnoid hemorrhage on the ventral surface of the brain and in the basal cisterns (Fig. 1).

### Histopathological findings

As a result of the light microscopic examination of Group 1 (Control), it was seen that the arterial wall included the intima, media and adventitia layers, which have a normal histological structure, from the inside to the outside.

**Figure 3:**
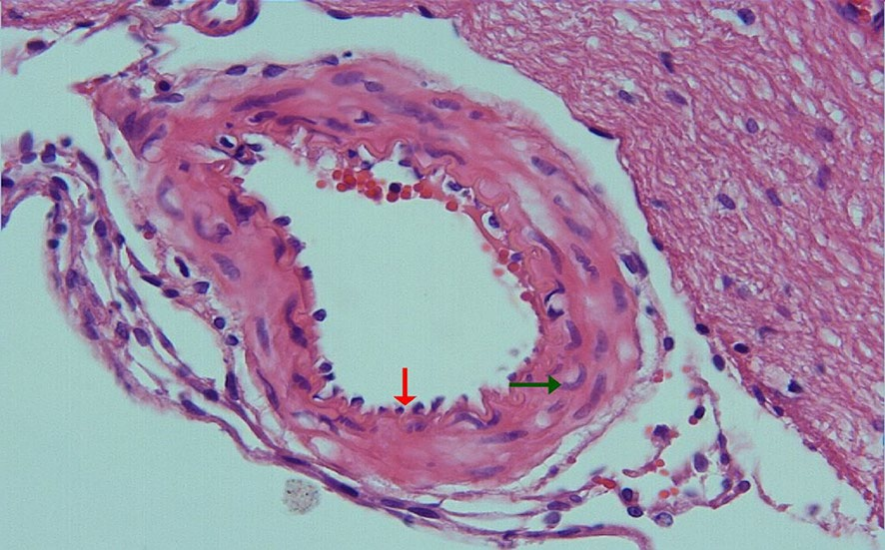
Group 2; arterial endothelial damage, prominent twisting-ondulation (red arrow) in the lamina elastica interna, contraction and vacuolization in the smooth muscle cells of the media layer (green arrow), H-E, × 40.

**Figure 4:**
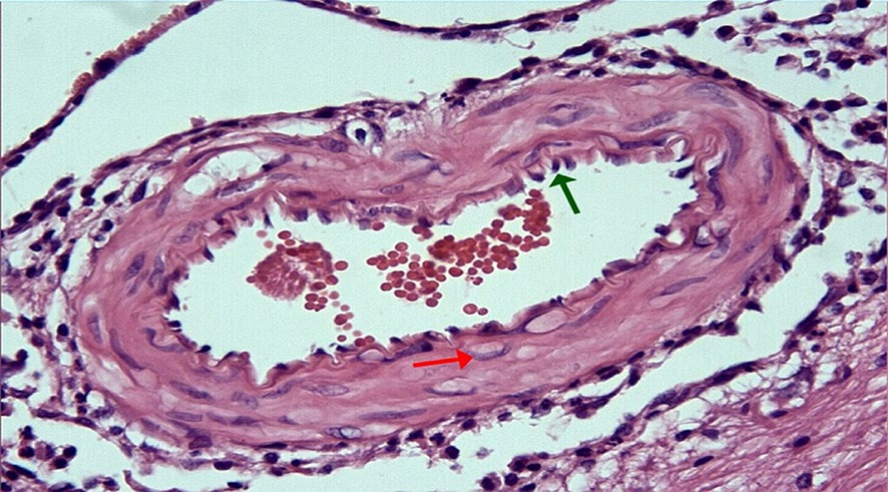
Group 2; significant curling-ondulation (green arrow) in the internasal artery lamina elastica, swelling and vacuolization in the smooth muscle cells of the media layer (red arrow), H-E, × 40.

In the arterial sections of group 2, protrusion of endothelial cells towards the lumen, intracellular edema in the perinuclear area, and separations from the underlying elastic lamina were observed in places. In the folds of the lamina elastica interna, the contraction of the smooth muscle cells of the media layer was increased and their length was shortened. Cytoplasmic vacuolization and swelling in smooth muscle cells were noted (Figs. 3, 4).

**Figure 5:**
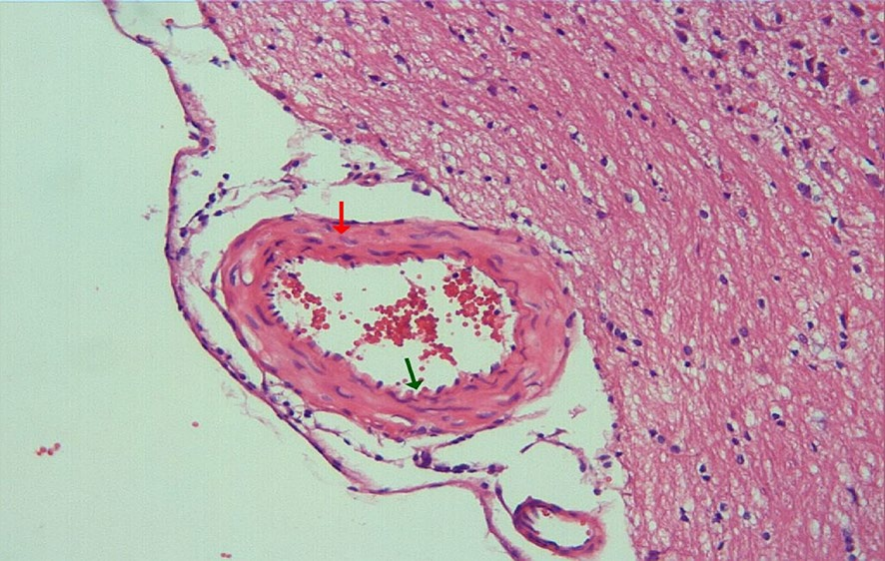
Group 3; minimal contraction of the smooth muscle cells of the arterial media layer (red arrow), moderately curved appearance in the artery lamina elastica interna (green arrow), H-E, × 20.

**Figure 6:**
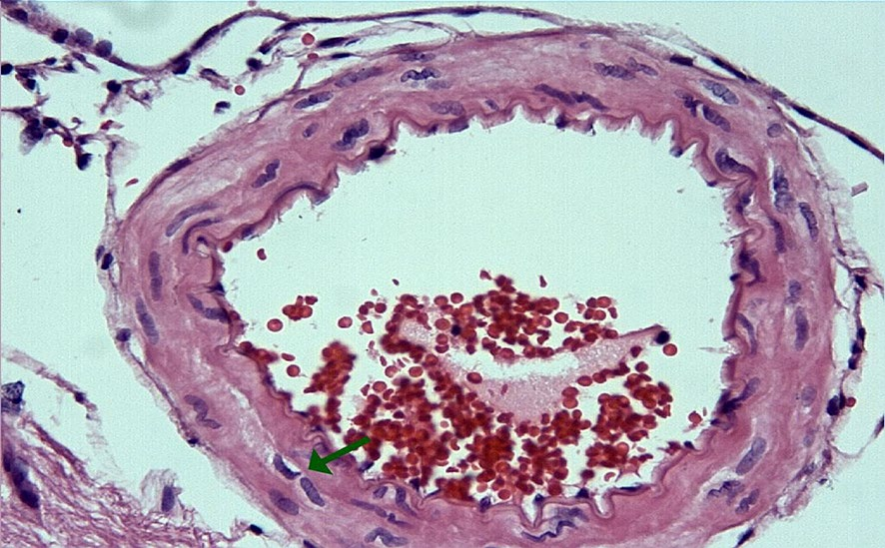
Group 3; contraction of smooth muscle cells in arterial media layer (green arrow), H-E, × 40.

In group 3, endothelial cells protruding towards the lumen, folded lamina elastica interna, and contraction in the smooth muscle cells of the media layer were observed in arterial sections, but these findings were less pronounced, and the lumen contour was more flat compared to the second group (Figs. 5, 6).

**Figure 7:**
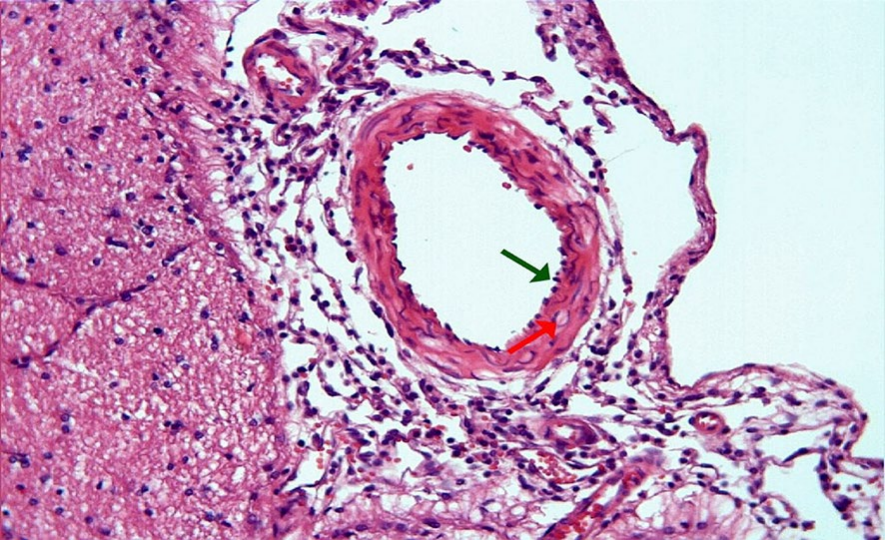
Group 4; contraction of smooth muscle cells in the arterial media layer (green arrow), minimal inflammatory cell infiltration in the perivascular area. H-E, × 20.

**Figure 8:**
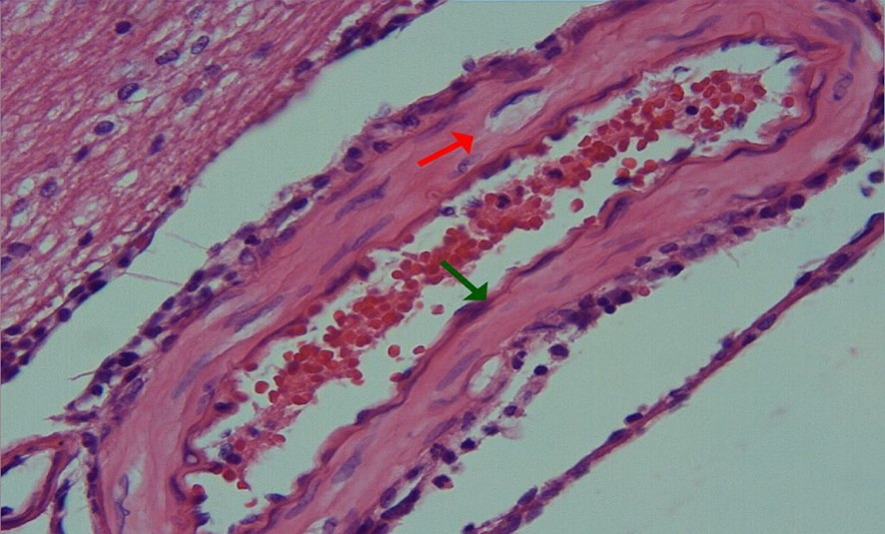
Group 4; minimal intracellular edema (red arrow) in smooth-looking endothelium (green arrow) and smooth muscle cells of the media layer. H-E, × 40.

Histological findings of vessel sections in group 4 (SAH + ALCAR 100 mg/kg) were similar to those in the third group. Endothelial cells were seen protruding from the luminal surface. In the smooth muscle cells of the media layer, the contraction was minimal and there was local intracytoplasmic edema. (Figs. 7, 8).

**Figure 9:**
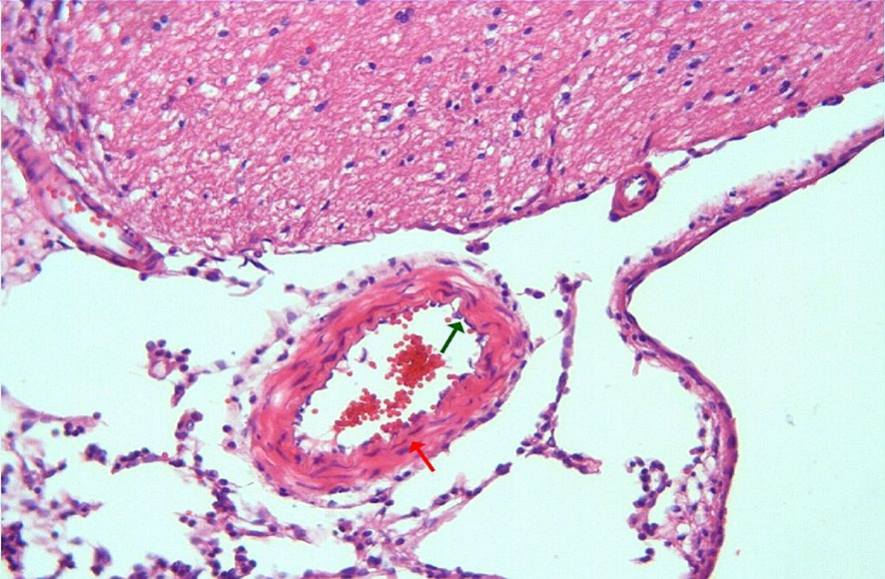
Group 5; protrusion towards the lumen in arterial endothelial cell nuclei (green arrow), contraction (red arrow) in smooth muscle cells of the media layer. H-E, × 20.

**Figure 10:**
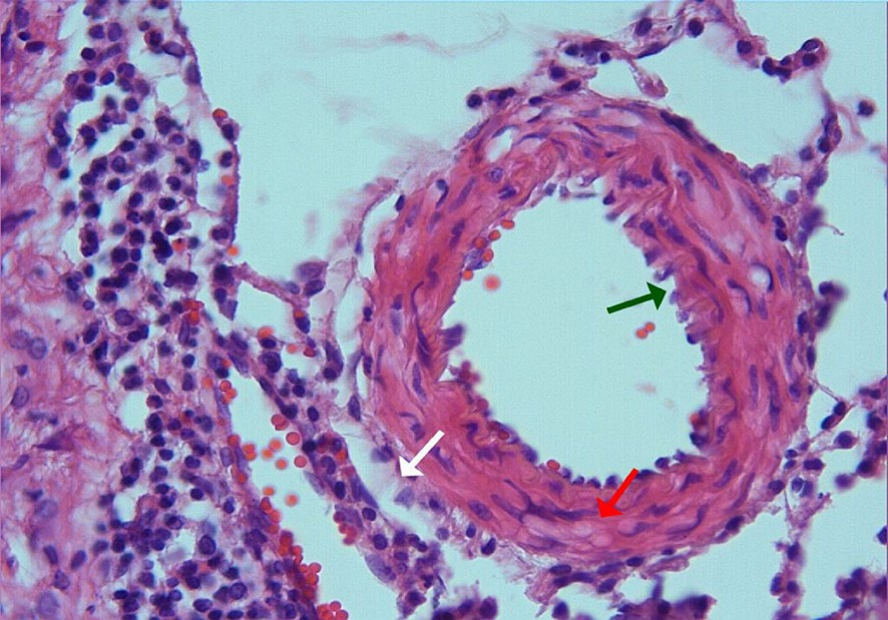
Group 5; protrusion towards the lumen in arterial endothelial cell nuclei (green arrow), contraction and vacuolization in media layer smooth muscle cells (red arrow), inflammatory cell infiltration in the perivascular area (white arrow). H-E, × 40.

In group 5 (SAH), it was determined that the nuclei of the endothelial cells protruded towards the lumen in the arterial sections, similar to the second group, the lamina elastica interna was quite convoluted, and there was a significant contraction in the smooth muscle cells of the media layer. Cytoplasmic vacuolization was noted in the smooth muscle cells (Figs. 9, 10).

A statistical difference was found between the mean lumen cross-sectional areas in all five groups. The mean lumen cross-sectional area was found to be higher in the control group when compared to the other groups. Moreover, there was a statistically significant difference between the control group and SAH group, SAH + SF group, and SAH + ALCAR 100 mg\kg group (p = 0.0408) (Table 1). On the other hand, there was no statistical difference between the SAH group, SAH + SF group, and SAH + ALCAR 100 mg\kg group (p > 0.05).

**Table 1:** Lumen cross-sectional area means and standard deviations by groups.

	Lumen cross-sectional area (µm^2^)			
	Minimum	Maximum	Median	Mean ± SD
Control (n = 7) (P = 0.0408)	55,011.66	129,122.48	74,599.4408	76,088.6964 ± 25,041.50544
SAH + SF (n = 6) (p < 0.005)	31,670.32	48,981.62	40,942.8270	40,985.5733 ± 6472.03359
SAH + ALCAR 50 mg\kg (n = 6) (p < 0.005)	36,210.16	111,249.54	65,376.5402	68,772.5296 ± 30,217.90492
SAH + ALCAR 100 mg\kg (n = 6) (p < 0.005)	32,158.93	74,251.23	47,111.3965	50,737.0302 ± 16,022.84597
SAH (n = 6) (p < 0.005)	26,911.44	91,824.63	40,427.9995	47,591.9022 ± 24,123.27345

A statistical difference was found between the mean vessel wall thickness in all five groups. The mean vessel wall thickness was found to be lower in the control group when compared to the other groups. Moreover, there was a statistically significant difference between the control group and SAH group, SAH + SF group, SAH + ALCAR 100 mg\kg group, and SAH + ALCAR 50 mg\kg group (p = 0.0267). There was no statistical difference between SAH group, SAH + SF group, SAH + ALCAR 50 mg\kg group and SAH + ALCAR 100 mg\kg group (p > 0.05) (Table 2).

**Table 2:** Mean and standard deviation of basilar artery wall thickness by groups.

	Basilar artery wall thickness (µm)			
	Minimum	Maximum	Median	Mean ± SD
Control (n = 7) (p = 0.0267)	197.26	260.44	223.2235	223.2235 ± 24.86788
SAH + SF (n = 6) (p < 0.005)	237.07	300.39	258.4981	263.7760 ± 27.32636
SAH + ALCAR 50 mg\kg (n = 6) (p < 0.005)	255.45	368.64	267.9204	289.0519 ± 43.72547
SAH + ALCAR 100 mg\kg (n = 6) (p < 0.005)	238.00	295.52	273.7340	268.0593 ± 21.98435
SAH (n = 6) (p < 0.005)	231.54	296.32	269.3850	264.3643 ± 23.55727

### Biochemical findings

Serum and tissue levels of L-Arginine, ADMA SDMA, and NO were measured by taking blood samples from all subjects in the groups before sacrification, and also samples taken from the parenchyma after sacrification. SDMA serum levels were higher in the control group when compared to the other groups. A statistically significant increase was found in the control group and SAH + SF group when compared with the other groups (p = 0.0007).

When comparing the tissue samples of ADMA with multiple variance analysis between groups; There was a statistically significant difference between SAH + ALCAR 50 mg\kg and SAH groups and Control, SAH + SF and SAH + ALCAR 100 mg\kg groups (p = 0.0015). ADMA level increased in SAH + ALCAR 50 mg\kg and SAH GROUPS. In addition, when the NO ratios in serum samples were compared with the analysis of multiple variances between groups, a statistically significant difference was found in the SAH + SF group compared to the other groups (p = 0.0016) NO serum level increased in the SAH + SF group.

## Discussion

Treatment of vasospasm after SAH and the results obtained are still among the topics discussed in the literature. It has been reported that the most important cause of mortality and morbidity after ruptured aneurysm is vasospasm^19^. The main factor in the development of vasospasm is the clot residues around the arterial structures.

Vasospasm was first demonstrated radiologically in 1951 and has subsequently been the subject of many studies. While radiological vasospasm was detected in 70% of patients with SAH, symptomatic vasospasm was observed in only 30–40%^20^. Spasmogens released by hemolysis of the periarterial clot are thought to be the underlying mechanism. Although it is thought that the most ideal research that can be done on this subject is on human cerebral arteries, in the study by Solomon et al.; it has been reported that rats are potential models for SAH studies. In the same study, they also found that vasospasm in the basilar artery showed the same characteristics as in humans^21^. The efficacy of metabolites in vasospasm has been investigated using different animal models, doses and forms^22^.

Rats were used in our study because they are easily available, inexpensive, easy to maintain, and their vasospasm processes are similar to humans. Although experimental SAH production is technically grouped under 3 headings, many methods have been developed based on these techniques. First technique is the collecting blood by puncturing the basilar artery or a large artery. The second technique is surgical dissection of the artery and insertion of autologous blood from another surrounding artery. Third and last is the most frequently used one in studies that the injection of autologous arterial blood into the subarachnoid space^23^. The common features of experimental vasospasm models, especially in rats, are that they do not develop neurological deficits. This is due to intensive collateral circulation. Also, as in humans, the adventitia layer of the arterial system in the subarachnoid space is not developed and the vasa vasorum is absent. Arteries are fed from CSF. The basilar artery was chosen for morphometric measurements in animal models because the posterior circulation is more developed in rats. The most obvious indicator of vasospasm is the reduction in basilar artery lumen diameter. It is also accompanied by changes in the cells of the internal elastic layer.

In our study, we used the bilateral bleeding model, which was used by Aladag et al. and most frequently used one in similar studies^16,24^. In this model, we made two nonheparinized autologous injections 48 h apart into the basal cisterns (via the cisterna magna). With this application, we aimed to provide better arterial vasoconstriction compared to a one-time blood injection.

We aimed to investigate the effect of Acetyl-L-Carnitine (ALCAR) on vasospasm in an experimental SAH model, which has many physiological effects in the body, and its main task is to transport long-chain fatty acids to the mitochondrial matrix. In our study, the first results obtained with ALCAR on vasospasm in the experimental SAH model. According to the results of our study, ALCAR prevented the formation and development of cerebral vasospasm. Our results were found to be consistent with Seckin H. et al.'s study investigating the effects of lamotrigine, an antiepileptic, on vasospasm in a rabbit experimental SAH model^25^. A significant increase in basilar artery lumen diameter and thinning of the basilar artery wall were detected in the treatment group compared to the SAH group which is compatible with literature^26,27^.

Coagulation occurs as a result of decrease in cerebral perfusion pressure and as a result, the ischemic process will begin. Subsequently, many intertwined reactions such as acute inflammation initiated by blood cells, free radical production, lipid peroxidation, vascular endothelial cell destruction, decreased NO, increased amount of endothelin, and intracellular calcium accumulation as a result of vascular cell membrane damage are indicators of how complex the event is. The most popular hypothesis is the development of vasospasm with the destruction of blood products^28^. The immediate onset of hemolysis after SAH and its continuation until the lysis and phagocytosis of erythrocytes attracted the attention of researchers, and as a result of studies, it was shown that the main substance responsible for vasospasm is oxyhemoglobin^29^. Accordingly, oxyhemoglobin will bind NO, and vascular contraction and vasospasm will develop as a result of the decrease in NO concentration^20^. Again, free iron in the environment will increase oxyhemoglobin and lipid peroxidation, and as a result, free radical production will be increased. Free radicals will disrupt the blood–brain barrier and the event will also result in endothelial damage^30^.

Studies in humans and animal models have since supported the notion of a multifactorial pathophysiology of delayed cerebral ischemia. Therefore, the main mechanisms and potential implication of therapies under investigation have shifted to the early brain injury, cortical spreading depolarizations, and neuro inflammation. Carnitine, a compound primarily involved in energy metabolism, has been studied for its potential impact on various neurological conditions. However, its specific effect on early brain injury, cortical spreading depolarizations (CSD), or neuro inflammation isn't extensively documented. Regarding early brain injury, some research suggests that carnitine might have a neuroprotective role due to its involvement in energy metabolism^31,32^. It's believed to help in maintaining mitochondrial function, potentially reducing cellular damage after injury^33^. In terms of cortical spreading depolarizations, which are implicated in conditions like migraine or traumatic brain injury, there's limited direct evidence on how carnitine specifically influences these events. Regarding neuro inflammation, there's emerging research indicating that carnitine might possess anti-inflammatory properties. It's suggested that it could modulate inflammatory responses in the brain, potentially reducing the impact of neuro inflammation on various neurological conditions^34^.

In our study, the fact that NO levels measured from serum samples were higher in groups with low lumen cross-sectional area was directly proportional to the severity of vasospasm. The more severe the vasospasm is (the lumen cross-sectional area will decrease), the higher the NO levels will increase to resolve this spasm. However, although the mean lumen cross-sectional areas were higher in the groups receiving ALCAR 50 mg\kg and ALCAR 100 mg\kg compared to the other groups, the fact that NO levels did not decrease at the same rate indicates that vasospasm was tried to be resolved by other mechanisms in the groups receiving treatment. Considering that the high NO level is caused by endothelial damage, it can be said that the endothelium is preserved in ALCAR groups. In our study, we wanted to investigate whether it is effective in solving vasospasm by using an antioxidant, ALCAR. Histopathologically and morphometrically, the mean lumen cross-sectional area was significantly different from the other groups in both groups receiving ALCAR. This shows that ALCAR can be tried as an alternative agent in the treatment of vasospasm.

## Conclusion

In light of all the findings, the antioxidant properties of ALCAR has positive effects on the prevention of vasospasm in the experimental SAH model. Potentially preventing vasospasm and reducing its clinical effects will directly contribute to the prognosis of SAH patients and increase the leverage of surgeons in the management process. But of course, more research on this subject will reveal clearer results.

## Data Availability

Data will be made available on reasonable request by corresponding author.
